# Development of Mono-Material Multilayer Light Barrier Films

**DOI:** 10.3390/polym17243279

**Published:** 2025-12-10

**Authors:** Rocío Ayelén Fuentes, Giacomo Foli, Roberta Di Carlo, Yanela Natalyn Alonso, Luciana Andrea Castillo, Matteo Minelli

**Affiliations:** 1 Planta Piloto de Ingeniería Química, PLAPIQUI (UNS-CONICET), Bahía Blanca 8000, Argentina; rfuentes@plapiqui.edu.ar (R.A.F.); yalonso@plapiqui.edu.ar (Y.N.A.); lcastillo@plapiqui.edu.ar (L.A.C.); 2 Departamento de Ingeniería Química, Universidad Nacional del Sur, Bahía Blanca 8000, Argentina; 3 Department of Civil, Chemical, Environmental and Materials Engineering (DICAM)—Alma Mater Studiorum, University of Bologna, 40131 Bologna, Italy; giacomo.foli2@unibo.it (G.F.); roberta.dicarlo5@unibo.it (R.D.C.)

**Keywords:** mono-material, multilayer films, polypropylene, composite, mineral particles, talc, kaolinite, light barrier properties

## Abstract

Mono-material multilayer polypropylene films were developed as light barrier structures through the incorporation of mineral-filled composite layers. Trilayer films with different layer arrangements were fabricated by thermocompression from polypropylene-based films containing 0, 1 and 5 wt.% of talc and kaolinite. A monolayer polypropylene film of equivalent total thickness was used as a control. Structural, thermal, mechanical, optical, and gas barrier properties were evaluated for all films fabricated. A well-defined trilayer structure was confirmed by SEM. FTIR analysis demonstrated negligible thermo-oxidation, with no thermal-degradation during processing. Improved thermal stability and a slight modification in crystallinity were evidenced by TGA and DSC, respectively. XRD revealed the predominance of the α-form crystalline phase and a preferential polymer crystal orientation associated with the particle presence. Regarding mechanical behavior, enhanced stiffness and tensile strength without loss of sealability or puncture resistance were observed. Trilayer films exhibited significantly reduced UV and visible light transmittance, while maintaining adequate translucency, making them suitable for photosensitive packaging applications. Gas permeabilities remained nearly unchanged, confirming that the barrier performances were preserved. Overall, these mono-material multilayer composites films offer a promising and recyclable alternative to conventional multi-material light barrier packaging, combining improved UV protection, mechanical robustness, and environmental compatibility.

## 1. Introduction

Packaging plays a key role in preserving product quality and extending shelf-life, particularly for those sensitive to light (UV and visible), oxygen, temperature, and humidity [1,2,3]. Light exposure, in the presence of oxygen, accelerates lipid photooxidation, leads to nutrient loss and deterioration of sensory attributes [4,5]. Therefore, selecting appropriate packaging materials is crucial, as they must fulfill the specific needs required by the product. Consequently, high barrier films have attracted significant interest in both research and industry, particularly for photosensitive products, as these alternatives offer enhanced product preservation [6,7].

Polyolefins such as polyethylene (PE) and polypropylene (PP), along with polyethylene terephthalate (PET) and polystyrene (PS), are widely used in packaging due to their ease of processing, lightweight, cost-effectiveness, and transparency [8,9]. However, monolayer polymer films can hardly provide all the properties required to protect and preserve photosensitive products. In multilayer systems, the final performance strongly depends on the processing method and the ability to control key variables that govern interlayer adhesion, polymer–polymer compatibility, as well as on the contribution of each layer to the overall structure. Such aspects are closely linked to the selected polymers, their physicochemical interactions, and the production scale [10]. Hence, the packaging industry often uses multilayer multi-material polymer films manufactured through conventional technologies, including coextrusion, lamination, and coating [10], to ensure the required high barrier properties for effective product preservation [11]. These technologies can be employed to combine different materials, such as aluminum or cardboard, requiring in certain cases the addition of a thin adhesive layer to ensure proper adhesion between the layers [12]. Specific requirements may depend on the packed product, and typically include gas and light barrier performance, as well as proper sealing. Gas barrier properties are particularly critical in food packaging, as they limit oxidation, control the internal atmosphere, and slow down ripening and spoilage processes. Key gases include oxygen, carbon dioxide and ethylene due to their direct role in oxidative degradation, microbial inhibition in modified atmosphere packaging (MAP), and fruit ripening, respectively [13,14]. Additionally, good optical and mechanical properties are crucial for flexible packaging [6].

In order to improve the light barrier in multilayer films, aluminum is commonly incorporated by coating or lamination, as it blocks UV and visible light effectively. Similarly, a paper layer can be added to enhance light protection while simultaneously providing rigidity and improving printability. Another common strategy to increase light barrier and attract consumers, is printing colorful and attractive designs on the packaging [15]. Nevertheless, the combination of different materials, or the use of adhesives and inks are not sustainable solutions. Conventional recycling methods by reprocessing cannot be employed for these packaging materials [16]. Several alternative approaches have been explored, including delamination, dissolution–reprecipitation, and compatibilization techniques [17,18]. However, these methods remain economically unfeasible, leading to the frequent disposal of multilayer films through landfilling (increasing environment passive) or incineration [19].

Regulatory and market pressures have recently accelerated the transition toward sustainable packaging. For example, the European Union objective of fully recyclable multilayer packaging by 2030 underscores the urgent need for circular-design strategies [20]. That shifts the material selection toward simpler, more homogeneous structures, compatible with conventional mechanical recycling. Consequently, “design for recycling”, or eco-design, has become a central principle, guiding the development of functional packaging that can be easily reintegrated into the production cycle [21].

Within this context, mono-material multilayer films based on polymer composites offer a promising route to combine high barrier performance with improved end-of-life options. These structures employ the same polymer matrix (usually PE or PP) in all layers, while their functionality can be tailored by incorporating fillers in low loadings (below 5 wt.%) [15]. Particularly, mineral particles such as talc, kaolinite, mica and calcium carbonate can enhance mechanical, thermal and barrier properties, simultaneously reducing production costs [22,23]. These fillers influence the final mechanical performance in two main ways: (i) by acting directly as rigid particles with specific characteristics such as shape, size, and modulus; and (ii) by modifying the crystallization behavior of the polymer matrix and, consequently, the molecular structure of the semicrystalline polymer [24].

Specifically, in semicrystalline polymers like PP and PE, certain inorganic particles can act as nucleating agents, modifying crystallinity, as well as type and crystal size, and consequently influencing their barrier performance [25]. Moreover, laminar mineral fillers such as talc and kaolinite can scatter and/or absorb light, thereby limiting light transmission [26,27,28,29], and protecting the product against photooxidation when well distributed and dispersed [30]. Furthermore, these films can be easily adapted to modern industrial production technologies and due to their low particle content, they can be recycled by conventional reprocessing. Recent advances in mono-material multilayer films have primarily focused on enhancing gas barrier performance while ensuring compatibility with mechanical recycling. Most studies report on PE-based multilayer structures in which barrier improvements are achieved through the incorporation of functional additives into specific layers. For instance, Wang et al. [31] produced LDPE multilayers by blown film extrusion, with the incorporation of KMnO_4_, pumice and NaCl, resulting in enhanced oxygen barrier properties and ethylene-absorption capability. Other blown LDPE systems formulated with green tea extract have demonstrated simultaneous improvements in light, water vapor and oxygen barrier properties, along with antioxidant activity [32]. Other PE-based multilayer films, developed by thermocompression, combine an antimicrobial agent (*S. cerevisiae*) and activated carbon, or zeolite as carriers to provide protection against microbial spoilage [33].

In contrast, developments in PP-based mono-material films have mainly relied on the addition of thin inorganic coatings as barrier layers. For example, multilayer configurations incorporating AlO_x_ as an internal barrier layer have shown substantial reductions in oxygen transmission rates [34,35]. Renoldi et al. [36] evaluated BOPP/PP structures containing an inorganic coating for semi-hard cheese packaging and reported improvements mainly in carbon dioxide retention. Overall, despite these advances, most reported efforts focus on oxygen-barrier improvement rather than light-shielding capabilities. Moreover, studies on PP-based mono-material multilayers incorporating mineral fillers remain limited, particularly those assessing a comprehensive analysis of optical, mechanical, and gas barrier properties. Such gap highlights the need for research exploring the potential of mineral-filled PP multilayers as recyclable light barrier materials.

The present work aims to develop mono-material multilayer films based on polypropylene composites acting as light barrier systems. Although industrial multilayer films are typically produced using the techniques mentioned above, thermocompression was selected in this study as a laboratory-scale method to isolate and evaluate the effects of layer configuration and particle incorporation on the barrier and mechanical properties [33,37,38]. The main objective is to analyze the influence of the presence of mineral particles (talc and kaolinite) and the multilayer configuration on the structural, mechanical, optical, and gas-barrier properties of the resulting films. Particular emphasis was devoted to the assessment of the potential of these films to combine effective UV and visible light protection with mechanical performance, contributing to the development of sustainable packaging materials for photo-sensitive products.

## 2. Materials and Methods

### 2.1. Materials

Monolayer films based on a commercial homopolymer polypropylene (PP) (Petrocuyo 1102 H; with a melt flow index of 1.8 g/10 min at 230 °C/2.16 kg) provided by PetroCuyo (Ensenada, Argentina), were used to develop multilayer mono-material films. Composite monolayer films contain commercial mineral particles. Specifically, films contained talc (T), supplied by Dolomita SAIC (Alta Gracia, Argentina) with a median particle size (D_50_) of 6 µm and kaolinite (K), provided by Piedra Grande SAMICA (Avellaneda, Argentina) with a D_50_ of 4 µm. PP monolayer films exhibited a crystallinity degree of 42.1 ± 0.1%, whereas that of the composite monolayer films increased to 45.5 ± 0.5%.

### 2.2. Preparation of Films

Three-layer films were obtained by thermocompression of composite films prepared with different mineral particles (talc and kaolinite) and particle concentrations (0, 1 and 5 wt.%). Thermocompression conditions were 180 °C and 1.96 MPa pressure for 30 s; this was followed by cooling the obtained films under pressure. These parameters were selected based on preliminary tests, aimed at ensuring adequate interlayer adhesion while preventing thermo-oxidative degradation during processing. Different three-layer configurations with respect to the order of the composite films were fabricated, always having one of the outer layers as PP (the one supposed to be in contact with the packaged product). Three-layer films were named as PP/PP (particle type) (particle concentration)/PP (particle type) (particle concentration), e.g., PP/PPT5/PPT1. In addition, control films (PP monolayer) with an equivalent thickness of three-layer films, were prepared.

### 2.3. Film Characterization

#### 2.3.1. Structural and Thermal Characterization

Control and trilayer film thickness were determined using a micrometer (Mitutoyo, Mitutoyo Corporation, Takatsu-ku, Kawasaki, Japan) at different locations. The presence of the trilayers were corroborated, on the cross-sectional area of films, by Scanning Electronic Microscopy (SEM) with a LEO EVO 40 XVP-EDS Oxford X-Max 50 (Carl Zeiss AG, Cambridge, UK). These studies were performed on several points of cryofractured films with a gold-coated surface, using an argon plasma sputter coater (PELCO 91000, Ted Pella, Redding, CA, USA).

Thermal stability of control and trilayer films and their total particle concentration were evaluated by thermogravimetric analysis (TGA) using a TGA 5500 (TA Instruments, New Castle, DE, USA). Samples were heated from 30 °C up to 700 °C at a constant rate of 10 °C/min in N_2_ atmosphere.

Possible thermo-oxidative degradation induced by processing was evaluated semi-quantitatively by the carbonyl index employing Fourier Transform Infrared Spectroscopy (FTIR) in a Thermo Nicolet Nexus spectrometer (Thermo Fisher Scientific, Waltham, MA, USA). Spectra were recorded directly on the films, in transmission mode over the range of 4000–400 cm^−1^, with a resolution of 4 cm^−1^ and 10 accumulated scans. Carbonyl index is defined as the ratio of the area under the band assigned to this group (1700–1800 cm^−1^) and a PP reference band, which is not affected by thermal degradation (2720 cm^−1^) [39].

Control and trilayer films were also analyzed by Differential Scanning Calorimetry (DSC) using a TA Instruments Discovery DSC (Discovery DSC, New Castle, DE, USA). Heating scans were carried out from 30 °C to 200 °C at 10 °C/min under nitrogen atmosphere. Peak identification and area integration were performed using Trios software (v 4.1.1.33073, 2016). Bulk degree of crystallinity (x_bc_) of the polymer phase was calculated based on DSC data:

(1) $$x_{bc}=100\cdot \frac{{\Delta H}_{m}}{{\Delta H}_{m}^{100}\cdot (1-m)}$$

where ${\Delta H}_{m}$ is the melting enthalpy, m represents particle mass fraction and ${\Delta H}_{m}^{100}$ is the theoretical melting enthalpy of 100% crystalline PP (207.1 J/g) [40].

Films crystal structure was analyzed by X-ray Diffraction (XRD), in order to evaluate a possible crystal orientation due to particle presence. Diffractograms were obtained in a Philips PW1710 X-ray diffractometer (Philips, Almelo, The Netherlands), provided with a tube, a copper anode, and a detector operating at 45 kV and 30 mA with 2θ ranging from 3 to 60°. They were performed on both outer layers of each trilayer, as well as on the two external faces of control film.

#### 2.3.2. Mechanical Properties

Tensile mechanical properties were assessed using a universal testing machine (Instron Model 3369, ITW company, Groton, MA, USA) equipped with a 1 kN load cell. Tests were performed at 23 °C with a crosshead speed of 10 mm/min until the samples fractured, in accordance with ASTM D882 [41]. Ten specimens from control layer and trilayer films were tested (five cut in one direction and five cut perpendicularly to it). From these tests, Young’s modulus (E), tensile strength (σ_u_) and elongation at break (ε_b_) were determined.

Thermo-sealing capacity tests were conducted following ASTM F88 [42] in the same universal testing machine. Five sealed specimens of control and trilayer films were prepared using an impulse-wire thermosealer making sure the inner PP layers were in contact during the sealing process.

Puncture resistance was evaluated using a hemispherical probe with a penetration rate of 25 mm/min under ASTM F1306 [43], testing five specimens from control and trilayer films, using a universal testing machine. For trilayer films, the inner PP layer was positioned facing the probe during the test.

#### 2.3.3. Light Barrier Properties

The light barrier capacity of control and trilayer films was assessed by means of a T60 UV-Vis spectrophotometer (TG Instruments, Earl Shilton, Leicestershire, UK), operating in transmittance mode over a wavelength range of 190–700 nm. Rectangular samples were cut and placed in a quartz cuvette (path length: 1 cm) for the analysis. For this purpose, transmittance values at 300 nm and 600 nm, corresponding to UV and visible regions, respectively, were specifically analyzed. In addition, haze (H %) in accordance with ASTM D1003 [44], whiteness index (WI) and yellowness index (YI) under ASTM E313 [45] and ASTM D1925 [46], respectively, were analyzed using a Hunter Lab UltraScan XE colorimeter (Hunter Associates Laboratory, Inc., Reston, Virginia, USA) and Universe software (v 4.10, Service pack 2, 2001).

#### 2.3.4. Gas Barrier Properties

Gas barrier properties were evaluated using a static permeation apparatus, at 23 °C and 0% relative humidity, according to ASTM D1434 [47] (constant volume method). Circular film samples with a diameter of 25 mm were tested, and their thickness was measured at different locations. Pure gas permeability ($P_{i}$) to oxygen, carbon dioxide and ethylene of the control and trilayer films was determined. The measurements were performed maintaining a 0.2 MPa pressure difference across the film, and the molar flux through the sample was determined from the pressure increase in the calibrated downstream chamber, applying the ideal gas law:

(2) $$P_{i}=J_{i}\frac{l}{∆p_{i}}=\frac{V_{d}}{A\;R\;T}\frac{l}{∆p_{i}}\frac{dp_{id}}{dt}$$

where $J_{i}$: molar density flux of component *i*, $∆p_{i}$: partial pressure difference in component *i* at the two sides of the film, l: film thickness, *A*: sample area exposed to gas, *R*: universal gas constant, *T*: absolute temperature, $V_{d}$: downstream volume and $\frac{dp_{id}}{dt}$: variation in downstream partial pressure of component *i* with time [48]. Permeability results are reported in the non-SI units Barrer (1 Barrer = 10^−10^ cm^3^ (STP cm/s cm^2^ cmHg)).

### 2.4. Statistical Analyses

All analyses were conducted in triplicate, unless otherwise specified, to ensure the reproducibility and reliability of the results. The mean values and standard deviations were calculated based on the experimental data obtained from the replicates. Statistical differences among the means were determined through a one-way analysis of variance (ANOVA), which allows for assessing whether the variations observed among groups are statistically significant. When significant differences were detected (*p* ≤ 0.05), Tukey’s multiple comparison test was applied to identify which specific means differed from each other. In the tabulated results, different lowercase letters are used to indicate statistically significant differences between groups: means sharing the same letter are not significantly different, while those with different letters differ at the 95% confidence level.

## 3. Results

### 3.1. Film Structural and Thermal Characterization

**Figure 1:**
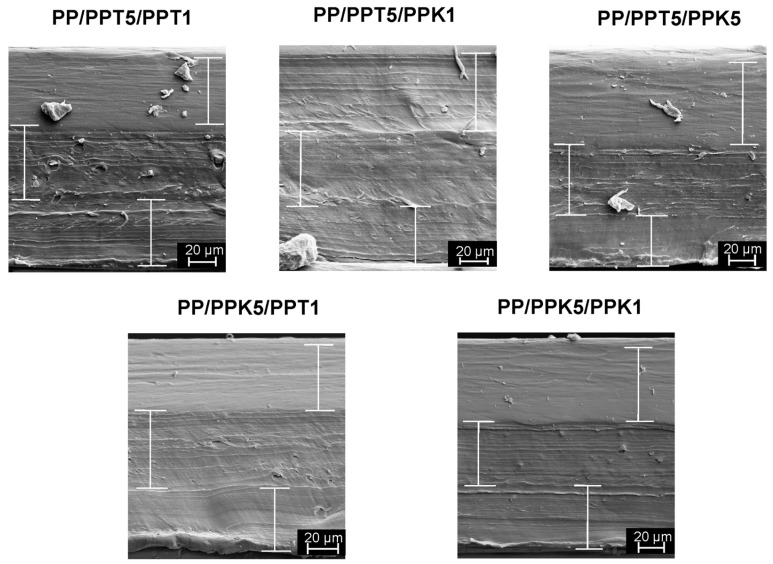
SEM micrographs of trilayer films (1000×). The vertical bars indicate the thickness of the individual layers.

The obtained films, including control monolayer film, present a mean thickness of 150 ± 11 µm. The presence of the trilayers was confirmed by SEM analysis, inspecting the cross-sectional area of films. In this sense, Figure 1 presents the SEM micrographs of three-layer structure of films, indicating a good interlayer adhesion, with no holes or gap between layers. Relevantly, the bottom layer corresponds to PP. Additionally, mineral particles can be identified, mainly in those layers with 5 wt.% fillers. Some of these particles are evident even though they are covered by a thin polymer skin.

**Figure 2:**
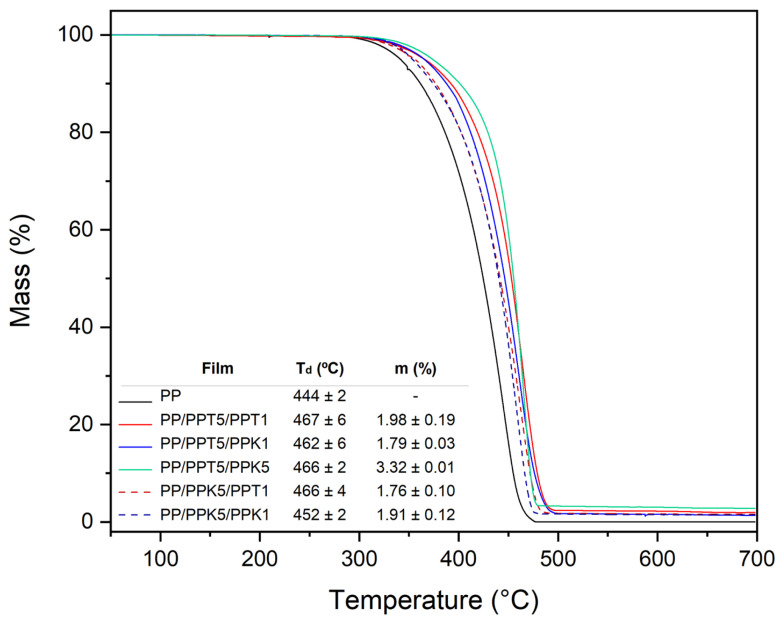
Mass percent versus temperature of control monolayer and trilayer films including degradation temperature values (T_d_) and total particle concentration (m %).

In order to determine total particle concentration and its influence on film thermal stability, Thermo-Gravimetric Analysis, TGA, was carried out. Figure 2 presents the dependence of the relative mass loss with temperature along with the decomposition temperature (T_d_). Specifically, T_d_ values were determined from the maximum decomposition peak in the derivative curve of thermogravimetric data. The analysis reveals that multilayer films filled with talc (PPT5 layer) present higher thermal stability in comparison with the films filled with kaolin (PPK5 layer) or the neat polymer (control PP monolayer film). The presence of the composite layer (with talc), in the trilayer configuration increases PP decomposition temperature by at least 23 °C, indicating an enhanced thermal resistance. Such behavior has been widely reported [25,49,50], and it can be attributed to their pronounced delamination capability, which enhances the barrier effect by limiting the release of volatile degradation products and consequently delaying the decomposition process [51]. Moreover, total particle concentration in trilayer films was verified from these curves, considering residual mass (m %) values at the end of polymer matrix degradation (Figure 2, inset). The residual masses are in accordance with the nominal total particle concentration theoretically determined in trilayer configuration from layer proportion and its filler concentration.

A qualitative assessment of possible thermo-oxidative degradation by film processing was carried out by FTIR spectroscopy. The presence of carbonyl groups, which may originate from the oxidation of tertiary carbons in the PP structure, serves as a reliable indicator of degradation. Therefore, the carbonyl index was calculated for all films, and reported in Table 1. These values are significantly lower than those reported by Salah et al. [52] and Espinosa et al. [53] for PP, suggesting negligible degradation levels.

Table 2 summarizes the bulk degree of crystallinity (X_bc_) and melting temperature (T_m_) obtained by Differential Scanning Calorimetry (DSC) for control monolayer and trilayer polypropylene-based films. The control monolayer film exhibited the lowest X_bc_ value (47.0%), which slightly increased upon the multilayer configuration, reaching up to 49.0% for the PP/PPT5/PPK5 film. That suggests the multilayer structure with composite layers promotes a modest increase in the overall crystallinity of the system. In this regard, the overall crystallinity obtained for all multilayer films is higher than the one that would be expected from the simple combination of the individual layers. Such behavior can be attributed to a possible recrystallization induced by the processing conditions and the presence of particles in the multilayer film. It is noteworthy that films containing a higher proportion of talc exhibit a greater increase in such property, which can be ascribed to the strong nucleating effect of talc. These results are consistent with the obtained T_m_ values which ranged between 161 and 163 °C, with small but statistically significant differences among the formulations, indicating that the multilayer configuration and the presence of particles only slightly influence the crystalline phase stability. The observed trend is in good agreement with previous findings by Espinosa et al. [53] and Meziane et al. [54], who demonstrated that the inclusion of mineral fillers does not significantly affect T_m_ in PP-based composites. In addition, Leong et al. [55] analyzed this effect by evaluating the crystallization behavior of PP in the presence of kaolin and talc, confirming that these mineral particles promote the formation of crystalline nuclei during cooling, thus inducing crystallization at higher temperatures, even though the overall crystalline fraction remains unchanged.

**Figure 3:**
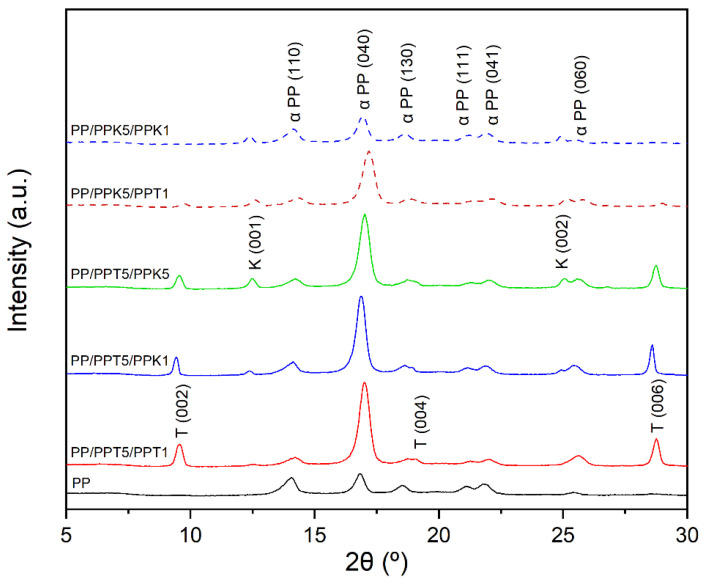
XRD spectra of control monolayer and trilayer films. Ref: T: talc, K: kaolinite, α PP: α crystal of polypropylene.

Polymer crystalline morphology is a key structural feature that strongly influences mechanical and barrier properties of composite films. In this sense, the relevant factors for composite films are the presence of filler, the loading, and particle morphology, as well as their orientation due to the monolayer processing. Particularly, in the case of laminar particles like talc and kaolinite, a macromolecular polymeric orientation is induced since particle surfaces offer active sites to crystal nucleation. Such behavior is observed in Figure 3, where diffractograms obtained by XRD are presented for all fabricated films. It is important to note that identical diffractograms were obtained regardless of which external surface of the films was exposed to the incident beam. Therefore, only one representative diffractogram per film is presented in Figure 3.

All diffractograms exhibit characteristic reflections of the stable monoclinic α-phase of PP associated with the (110), (040), (130), (111), (041), and (060) planes. The presence of talc is confirmed by its characteristic reflections at (002), (004), and (006) [56], while kaolinite is identified through its (001) and (002) planes [57]. Control monolayer film exhibited higher intensity in the (110) plane, while the (040) plane exhibited lower intensity compared to the trilayer films. The change in peak intensity suggests a preferential orientation of the b-axis of the PP crystals in (040) consistent with the alignment of laminar particles whose basal surfaces are parallel to the film surface. That can be attributed to monolayer processing and ulterior thermocompression to obtain multilayer configuration. The trend is more pronounced on trilayer films that contain a higher talc proportion in the formulation, in line with DSC results, indicating that the presence of talc particles could have a more pronounced nucleating effect than kaolinite [55].

### 3.2. Films’ Mechanical Properties

Mechanical properties of packaging films are crucial to ensure product protection during transport and storage. To fulfill these requirements, films must exhibit adequate performance to withstand external forces and mechanical stress. In polymer composite films, mechanical properties are influenced not only by the intrinsic characteristics of the polymer matrix but also by the presence of fillers and their possible influence on crystallinity [58]. Table 3 presents the main mechanical properties of control monolayer and trilayer films.

Since films were obtained by thermocompression that contributes to obtaining a uniform film structure, mechanical properties measured for the two directions were similar. In addition, during mechanical tests, no separation was observed between layers at failure for trilayer films, corroborating the good adhesion between different layers.

Table 3 shows that the trilayer configuration and the presence of particles led to an increase in Young’s modulus (E) compared to the control monolayer film. Although the differences among trilayer formulations were not statistically significant, if mean values are considered, the effect of talc particles is more significant on E values (i.e., PP/PPT5/PPT1) than when only kaolinite particles (i.e., PP/PPK5/PPK1) are present in trilayer films. The behavior can be associated with multiple reinforcing mechanisms for which different factors have to be accounted for: first, the intrinsic rigidity of particles that contributes to mechanical reinforcement of the PP matrix (E_talc_: 41.6 GPa [59] and E_kaolinite_: 6–12 GPa [60]); additionally, the presence of mineral particles, mainly talc, which modify the crystalline structure, enhancing material stiffness. Thus, a partial replacement of PP by more rigid fillers also restricts the mobility and deformability of the matrix by the introduction of a mechanical restraint. These findings are consistent with previous studies that reported an increment in Young’s modulus in PP composites containing talc [55,61].

Regarding the tensile strength, it is observed that the trilayer films show an improved mechanical resistance compared to the control monolayer film. In particular, both elastic modulus and tensile strength are consistently higher for all trilayer configurations, indicating increased stiffness and an enhanced ability of the material to withstand applied stresses before failure. That indicates a good filler–matrix interaction, associated with the filler laminar morphology. Particles having this morphology and large aspect ratios allow a higher filler wettability by the matrix, avoiding the generation of microvoids between particles and matrix. The incorporation of mineral particles enhances the stiffness and load-bearing capacity of the films by promoting stress transfer from the polymer matrix to the rigid inorganic particles. Due to their laminar morphology, talc and kaolinite act as effective reinforcing agents that restrict the mobility of the PP chains and hinder plastic deformation under tensile load [55]. Moreover, the increment in σ_u_ values enhances further the interfacial adhesion between the layers.

Elongation at break values for all multilayer films were found to be all above 5%, indicating that none of the formulations experienced brittle fracture in the test conditions [62]. Statistically significant differences (*p* < 0.05) in ductility were observed between control monolayer and composite trilayer configuration. Such decrease in elongation may be attributed to the increased stiffness imparted by the presence of particles, which restricts polymer chain mobility and introduces stress concentration points within the matrix. The behavior is consistent with the reported for polymer composites, where improvements in Young’s modulus are often accompanied by diminished elongation at break, particularly when rigid fillers are used [63].

Ensuring effective thermo-sealing is essential for multilayer films for packaging applications. In this study, all the composite films fabricated in this work demonstrated successful thermo-sealing capability, regardless of the type or concentration of mineral particles incorporated into the multilayer structure. Seal strength, defined as the maximum force required to separate two previously heat-sealed films, is the key parameter in evaluating packaging integrity. As shown in Table 3, no statistically significant differences were observed among the different formulations. The result is attributed to the fact that sealing occurred between the inner PP layers in all cases, confirming thus the quality of the three-layer structure. Consequently, appreciable variations in seal strength are not observed, even when mineral fillers are present in other layers. In terms of the failure mode, according to ASTM F88 standard, all specimens exhibited ductile failure, characterized by elongation followed by rupture within or near the sealed region indicating cohesive rather than adhesive failure, and further confirming effective sealing and a good adhesion between layers.

Puncture resistance is also critical for flexible packaging materials. In this sense, any loss of structural integrity can compromise barrier performance allowing the inflow or outflow of gases, moisture, or contaminants and consequently, shortening the shelf life of the product. In this study, puncture resistance was evaluated to assess the structural robustness of multilayer films and to examine the influence of mineral fillers and trilayer configuration on their ability to withstand localized mechanical stress. The results, presented in Table 3, show that puncture force values of trilayer films are comparable to those of the control monolayer film, with no statistically significant differences among the formulations (*p* > 0.05). That suggests the presence of talc and kaolinite at the studied concentrations, and the trilayer configuration did not affect the film resistance to puncture. The comparable performance across all samples indicates that filler incorporation, in the evaluated multilayer configurations, preserved the mechanical integrity of the films under localized deformation. Similar values were also expected, considering that, due to the processing, the particles are localized perpendicularly to the direction of the force during the puncture test. Thus, the contribution of mineral particles and trilayer configuration to puncture resistance was minimal under the applied test conditions. Moreover, no signs of interfacial delamination were observed after testing, confirming good adhesion between layers and the structural stability of the multilayer configuration.

### 3.3. Films Barrier Properties

#### 3.3.1. Films’ Light Barrier Properties

**Figure 4:**
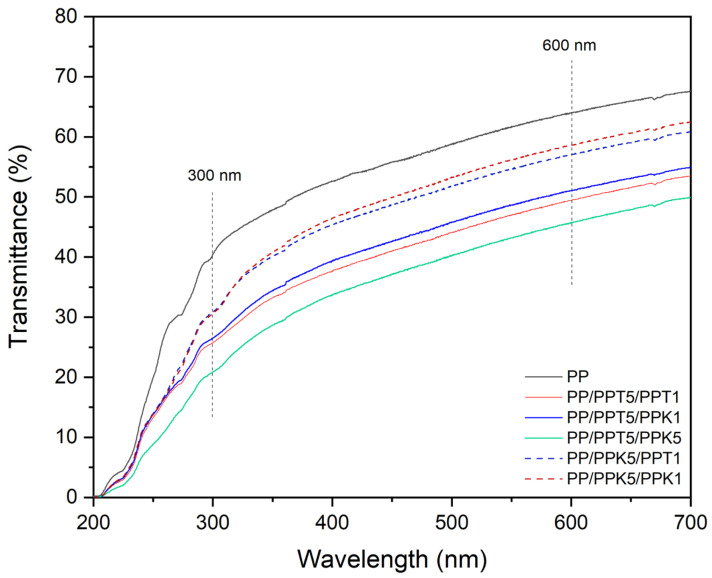
Transmittance spectra of control monolayer and trilayer films.

Light barrier properties are critical to reduce photo-oxidation and extending shelf-life of products, so the light transmittance of composite trilayer films at 300 nm in the UV range and 600 nm in the visible range was inspected. As shown in Figure 4, trilayer configuration containing mineral particles as fillers was more effective in enhancing light barrier performance. Regardless of the filler type, light barrier capacity increased with higher filler concentrations. Particularly, films containing a PPT5 layer further reduced light transmittance. The PP/PPT5/PPK5 formulation exhibited significant enhancement, reducing light transmittance by 31.2% at 600 nm (visible light) and a strong reduction by almost 50% at 300 nm (UV) with respect to control monolayer film. In this sense, mineral particles with laminar morphology, such as talc and kaolinite, can scatter and/or absorb light, preserving film translucency [26,27,28,29]. These fillers could also act as passive light barriers and modify polymer crystallinity during material processing, especially in semicrystalline polymers. The presence of crystals and mineral particles contributes to light scattering, creating a dual barrier effect. Such a synergistic mechanism and possible contribution of trilayer configuration significantly reduce light transmittance, enhancing light barrier properties of multilayer films [26].

Notably, the developed trilayer composite films combine excellent UV-barrier properties with good translucency in the visible region. It proceeds from the transmittance obtained values for all films developed (control and trilayer films), ranging between 10% and 80% according to Guzmán-Puyol et al. [8]. Such a balance is highly advantageous for packaging applications: on the one hand, blocking UV radiation helps to prevent photo-oxidative degradation, on the other hand, maintaining translucency in the visible range preserves product visibility, a key attribute for consumer acceptance [64]. Consequently, the developed mono-material multilayer films represent a promising alternative for applications requiring both strong UV protection and visual appeal, without compromising recyclability.

To gain further insight into the visual appearance and light transmission behavior of the developed films, optical measurements were performed, including haze, whiteness index (WI), and yellowness index (YI). Such parameters provide valuable information on how light interacts with the polymer matrix and the incorporated fillers, influencing both the transparency and the perceived color of the films. Haze quantifies the degree of light scattering, thus indicating the level of translucency, while WI and YI describe the brightness and color tone of the samples, respectively. The evaluation of these properties is essential to understand the effect of mineral fillers and multilayer configurations on the appearance and functional optical performance of the materials. Table 4 summarizes the optical properties of control and multilayer films, including haze, WI, and YI. The monolayer PP film exhibited a haze value of 32.6%, indicating a partially translucency.

The incorporation of mineral-filled layers led to a significant increase in haze, reaching values between 43.9% and 56.1%, depending on the layer configuration. In particular, the PP/PPT5/PPK1 and PP/PPT5/PPK5 films showed the highest haze values (52.6% and 56.1%, respectively), which can be attributed to enhanced light scattering caused by the presence of mineral particles and interfacial refractive index mismatches between layers. Correspondingly, the WI values decreased slightly with increasing haze, whereas YI exhibited a moderate rise, especially for the multilayer systems containing talc as filler, suggesting a subtle color shift toward a warmer tone.

#### 3.3.2. Films’ Gas Barrier Properties

**Figure 5:**
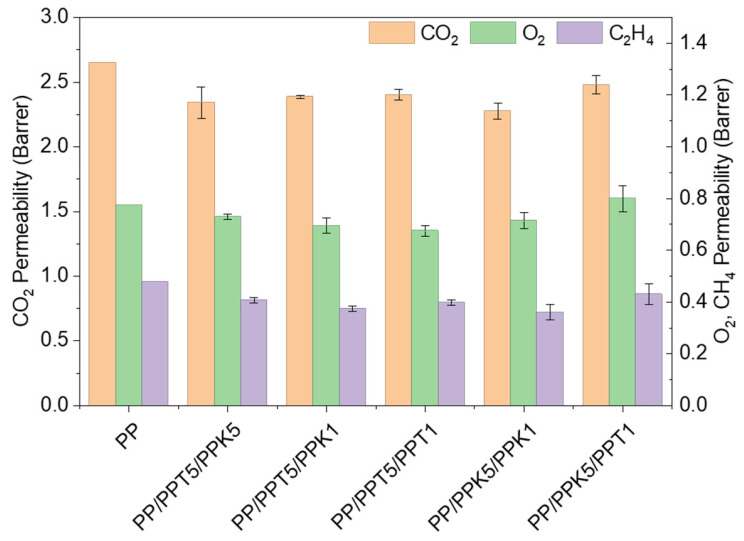
CO_2_, O_2_ and C_2_H_4_ permeability (Barrer) at 23 °C in multilayer films.

Understanding and optimizing gas permeation characteristics in multilayer films is essential for ensuring product protection and enhancing packaging performance. Gas permeability (*P*) in polymeric systems is governed by the solution–diffusion model [65]:

(3) P=D×S

where *D* is the diffusivity and *S* is the solubility of the penetrant gas. The equation highlights how both kinetic and thermodynamic factors contribute to gas transport through the material. As shown in Figure 5, carbon dioxide (CO_2_) exhibited the highest permeability, followed by oxygen (O_2_) and ethylene (C_2_H_4_). Compared to the control film, PP-based trilayer films showed small but systematic reductions in permeability from 5% to 25%, where the largest variations were recorded for CO_2_ and C_2_H_4_, while O_2_ permeability remained nearly unchanged.

Time-lag measurements of diffusivity revealed the following trend: $D_{O_{2}}>D_{CO_{2}}>D_{C_{2}H_{4}}$ (Table 5). This ordering correlates well with the molecular size of gases, if evaluated as molar volume at the critical point, O_2_ being the smallest and C_2_H_4_ the larger penetrant. As expected, CO_2_ and C_2_H_4_ exhibited higher solubility in the polymer phase due to their greater condensability and affinity for the non-polar polymer matrix, whereas O_2_ showed faster diffusion but lower solubility. Consequently, the observed permeability reflected the combined and gas-specific contribution of these two effects.

The incorporation of talc and kaolinite as fillers within the polymeric matrix is expected to increase the tortuosity of the diffusive pathway, as these layered, plate-like particles act as impermeable obstacles, forcing gas molecules to take longer, more complex paths through the membrane. However, despite the large aspect ratio, low filler loading reduced the extent of this effect, leading to modest reductions in permeability, consistent with the small differences in the degree of crystallinity highlighted by the DSC analysis, upon the addition of fillers. Therefore, the multilayer configuration slightly enhanced the barrier performance of monolayer films while preserving their suitability for multilayer packaging applications.

## 4. Conclusions and Perspectives

Mono-material multilayer PP-based films containing talc- and kaolinite-filled layers were fabricated by thermocompression, achieving well-adhered structures without interfacial defects. The presence of mineral particles slightly modified polymer crystallinity and significantly enhanced thermal stability, particularly when talc was incorporated. The multilayer configuration contributed to improved stiffness and tensile strength while preserving sealability and puncture resistance, demonstrating the mechanical integrity of the system. Optical analysis revealed a notable reduction in UV and visible light transmittance together with increased haze, indicating that mineral fillers, such as talc and kaolinite, effectively enhanced light scattering and barrier performance. Gas permeability was preserved, revealing that the addition of fillers did not compromise gas barrier properties. Therefore, based on the obtained results, it is possible to state that the developed multilayer films combine an excellent light-blocking capacity and high mechanical robustness, positioning them as promising candidates for sustainable light barrier packaging applications. Future work should focus on the process optimization and scale-up of the fabricated mono-material multilayer films to enable their processing at an industrial level. In particular, the transition from thermocompression-based laboratory prototypes to continuous processes, such as melt compounding followed by blown-film coextrusion, would allow assessing the robustness of the materials under realistic manufacturing conditions. Optimization of key processing parameters, including melt temperature profile, die and barrel temperatures, shear rate, bubble stability, draw-down ratio, and cooling conditions, will be useful to ensure stable and consistent film barrier and mechanical properties during high-throughput production. Simultaneously, reprocessing trials could be carried out to evaluate property retention after multiple recycling cycles, thereby validating the circularity benefits associated with the mono-material design. Together, these research directions will support the large-scale implementation of these packaging materials, and for the assessment of the recyclability performance and long-term stability of the proposed multilayer structures.

## Figures and Tables

**Table 1:** Carbonyl Index for control monolayer and trilayer films.

Film	Carbonyl Index
PP	N.D.
PP/PPT5/PPT1	0.018 ± 0.002
PP/PPT5/PPK1	0.030 ± 0.002
PP/PPT5/PPK5	0.033 ± 0.001
PP/PPK5/PPT1	0.020 ± 0.001
PP/PPK5/PPK1	0.022 ± 0.002

Ref: N.D.: not detected.

**Table 2:** Bulk crystallinity degree (X_bc_) and melting temperature (T_m_) obtained by DSC for control monolayer and trilayer films.

Film	X_bc_ (%)	T_m_ (°C)
PP	47.0 ± 0.4 ^a^	161.9 ± 0.1 ^a^
PP/PPT5/PPT1	47.9 ± 0.1 ^b^	163.1 ± 0.5 ^b^
PP/PPT5/PPK1	48.5 ± 0.2 ^c^	163.0 ± 0.2 ^b^
PP/PPT5/PPK5	49.0 ± 0.6 ^c^	162.7 ± 0.5 ^b,c^
PP/PPK5/PPT1	48.3 ± 0.6 ^b,c^	162.4 ± 0.1 ^c^
PP/PPK5/PPK1	48.8 ± 0.3 ^c^	161.4 ± 0.3 ^d^

Different superscript letters (a, b, c, d) in each column indicate significantly different values (*p* < 0.05).

**Table 3:** Mechanical properties of control monolayer and trilayer films.

Film	Tensile Properties			Thermosealing Strength (N)	Puncture Resistive Force (N)
	E (MPa)	σ_u_ (MPa)	ε_b_ (%)		
PP	1430 ± 42 ^a^	30.2 ± 1.2 ^a^	10.8 ± 1.0 ^a^	76.5 ± 6.7 ^a^	35.0 ± 2.8 ^a^
PP/PPT5/PPT1	1779 ± 139 ^b^	33.2 ± 1.6 ^b^	7.0 ± 0.5 ^b^	76.9 ± 6.3 ^a^	36.2 ± 1.8 ^a^
PP/PPT5/PPK1	1748 ± 82 ^b^	33.8 ± 2.4 ^a,b^	7.7 ± 0.8 ^b^	75.3 ± 6.6 ^a^	36.5 ± 2.6 ^a^
PP/PPT5/PPK5	1761 ± 162 ^b^	34.1 ± 2.3 ^b^	7.8 ± 0.9 ^b^	75.1 ± 4.8 ^a^	35.4 ± 1.7 ^a^
PP/PPK5/PPT1	1763 ± 65 ^b^	34.5 ± 1.9 ^b^	8.3 ± 0.5 ^b^	73.7 ± 5.5 ^a^	35.9 ± 3.7 ^a^
PP/PPK5/PPK1	1678 ± 74 ^b^	30.8 ± 1.8 ^a,b^	8.3 ± 0.4 ^b^	77.8 ± 3.4 ^a^	35.2 ± 3.7 ^a^

Different superscript letters (a, b) in each column indicate significantly different values (*p* < 0.05).

**Table 4:** Optical properties of control and trilayer films.

Film	Haze (%)	WI	YI
PP	32.6 ± 0.7 ^a^	85.2 ± 0.1 ^a^	1.8 ± 0.0 ^a^
PP/PPT5/PPT1	43.9 ± 0.5 ^b^	79.4 ± 0.2 ^b^	3.4 ± 0.1 ^b^
PP/PPT5/PPK1	52.6 ± 1.2 ^c^	77.9 ± 0.0 ^c^	3.8 ± 0.0 ^c^
PP/PPT5/PPK5	56.1 ± 0.7 ^d^	76.7 ± 0.2 ^d^	4.1 ± 0.0 ^d^
PP/PPK5/PPT1	43.5 ± 1.7 ^b^	80.9 ± 0.2 ^e^	2.9 ± 0.0 ^e^
PP/PPK5/PPK1	44.3 ± 0.9 ^b^	81.9 ± 0.3 ^f^	2.6 ± 0.0 ^f^

Different superscript letters (a, b, c, d, e, f) in each column indicate significantly different values (*p* < 0.05).

**Table 5:** Diffusion and solubility coefficients for O_2_, CO_2_ and C_2_H_4_ in multilayer films.

Film	D × 10^−9^ [cm^2^/s]			S × 10^−2^ [cm^3^(STP)/(cm^3^ × atm)]		
	O_2_	CO_2_	C_2_H_4_	O_2_	CO_2_	C_2_H_4_
PP	95.7 ± 0.9	50 ± 1	5.6 ± 0.1	6.2 ± 0.6	40 ± 1	65 ± 1
PP/PPT5/PPK5	111 ± 12	53 ± 9	4.9 ± 0.1	5.2 ± 0.3	35 ± 7	64 ± 0.2
PP/PPT5/PPK1	934 ± 5	50 ± 3	5.4 ± 1.3	5.7 ± 0.5	37 ± 2	55 ± 14
PP/PPT5/PPT1	100 ± 8	53 ± 5	5.2 ± 0.7	5.2 ± 1.4	34 ± 5	59 ± 5
PP/PPK5/PPK1	93 ± 21	47 ± 9	4.9 ± 0.5	5.9 ± 1.1	38 ± 8	56 ± 1
PP/PPK5/PPT1	119 ± 4	55 ± 1	4.3 ± 2.0	5.2 ± 0.1	35 ± 3	85 ± 28

## Data Availability

Data will be made available on request.

## Abbreviations

The following abbreviations are used in this manuscript:

UV	Ultraviolet
PE	Polyethylene
PP	Polypropylene
PET	Polyethylene terephthalate
PS	Polystyrene
MAP	Modified Atmosphere Packaging
wt.%	Weight Percent
KMnO_4_	Potassium Permanganate
NaCl	Sodium Chloride
*S. cerevisiae*	*Saccharomyces cerevisiae*
AlOx	Aluminum Oxide
BOPP	Bioriented Polypropylene
T	Talc
K	Kaolinite
D_50_	Particle size at the cumulative particle size distribution percentage of 50%.
SEM	Scanning Electronic Microscopy
TGA	Thermogravimetric Analysis
FTIR	Fourier Transform Infrared Spectroscopy
DSC	Differential Scanning Calorimetry
X_bc_	Bulk Degree of Crystallinity
XRD	X-ray Diffraction
E	Elastic Modulus
σ_u_	Tensile Strength
ε_b_	Elongation at Break
H	Haze
WI	Whiteness index
YI	Yellowness index
P_i_	Pure gas permeability
ANOVA	One-way analysis of variance
T_d_	Decomposition temperature
m %	Residual mass
T_m_	Melting Temperature
O_2_	Oxygen
CO_2_	Carbon Dioxide
C_2_H_4_	Ethylene
